# The Hospitalization Cost of Pediatric *Staphylococcus aureus* Bacteremia

**DOI:** 10.1093/jpids/piaf114

**Published:** 2025-12-19

**Authors:** Keerthi Anpalagan, Christopher C Blyth, Jonathan R Carapetis, Anita J Campbell, Asha C Bowen, Jeffrey W Cannon

**Affiliations:** School of Medicine, University of Western Australia, Perth, WA, Australia; Wesfarmers Centre of Vaccines and Infectious Disease, The Kids Research Institute Australia, Perth, WA, Australia; School of Medicine, University of Western Australia, Perth, WA, Australia; Wesfarmers Centre of Vaccines and Infectious Disease, The Kids Research Institute Australia, Perth, WA, Australia; Department of Infectious Diseases, Perth Children’s Hospital, Perth, WA, Australia; Department of Microbiology, PathWest Laboratory Medicine WA, QEII Medical Centre, Perth, WA, Australia; School of Medicine, University of Western Australia, Perth, WA, Australia; Wesfarmers Centre of Vaccines and Infectious Disease, The Kids Research Institute Australia, Perth, WA, Australia; Department of Infectious Diseases, Perth Children’s Hospital, Perth, WA, Australia; School of Medicine, University of Western Australia, Perth, WA, Australia; Wesfarmers Centre of Vaccines and Infectious Disease, The Kids Research Institute Australia, Perth, WA, Australia; Department of Infectious Diseases, Perth Children’s Hospital, Perth, WA, Australia; School of Medicine, University of Western Australia, Perth, WA, Australia; Wesfarmers Centre of Vaccines and Infectious Disease, The Kids Research Institute Australia, Perth, WA, Australia; Department of Infectious Diseases, Perth Children’s Hospital, Perth, WA, Australia; School of Medicine, University of Western Australia, Perth, WA, Australia; Wesfarmers Centre of Vaccines and Infectious Disease, The Kids Research Institute Australia, Perth, WA, Australia

**Keywords:** *Staphylococcus aureus* bacteremia, costing, hospitalization, children, economic burden

## Abstract

**Background:**

*Staphylococcus aureus* bacteremia (SAB) is the most common cause of childhood sepsis contributing to pediatric intensive care unit admission. The cost of adult SAB hospitalization is well described globally, but limited costing information is available for children. To bridge this knowledge gap, we investigated the cost of hospitalization in children with SAB in Australia.

**Methods:**

An economic analysis of hospitalization costs involving children aged ≤18 years with SAB admitted to Perth Children’s Hospital (PCH) between January 2017 and December 2018 was completed. Children were identified from the Invasive *Staphylococcus aureus* Infections and Hospitalisations (ISAIAH) cohort, a prospective multicenter study of pediatric SAB in Australia and New Zealand. The primary measure was mean hospitalization cost of community-onset SAB, overall and stratified by key variables, with 95% CIs calculated by bootstrapping.

**Results:**

There were 61 patients with SAB admitted to PCH. Fifty-six patients had community-onset SAB. The mean hospitalization cost per patient with community-onset SAB was A$53 037 [95% CI $44 623-$61 452]. The annual total cost was A$1 485 058. Hospital-onset SAB costs were significantly higher than community-onset SAB costs, and there was no difference in hospitalization costs by antibiotic susceptibility profile for community-onset SAB. The total cost of pediatric hospitalization for community-onset SAB within the ISAIAH cohort was estimated to be over A$9 million.

**Conclusion:**

This study provides the first in-depth analysis of the cost of hospitalization for SAB in children. This study suggests that the economic burden of SAB in children is substantial, and prevention and treatment strategies should remain focused on *S. aureus* as a whole.

## INTRODUCTION

*Staphylococcus aureus* bacteremia (SAB) is a serious, life-threatening bacterial infection with a high incidence among children and adults (8.56/100 000 children per year and 14.11/100 000 adults per year in Australia).^1^^,^^2^ In the latest World Health Organisation Bacterial Priority Pathogen List (2024), *S. aureus* is classified as a high-priority, antimicrobial-resistant (AMR) pathogen due to its substantial disease burden.^3^ Due to the significant variability in clinical severity and complexity of management, SAB among children leads to a corresponding variation in hospitalization costs. Establishing and analyzing the hospitalization costs of SAB among children improves our understanding of the economic impact SAB has on our healthcare system. It is also critical for evaluating the cost-effectiveness of new interventions, including new models of care, treatments, and prevention strategies. Baseline data will assist with the analysis of cost-effectiveness evaluation of new interventions that are currently being trialed, such as those in the *Staphylococcus aureus* Network Adaptive Platform (SNAP)^4–7^ trial and SNAP substudies,^8^ which are evaluating a range of interventions in children and adults with SAB.

Despite the heavy burden of SAB across the life course, progress in vaccine development for *S. aureus* has been stagnant, and the pipeline to phase 3 and 4 clinical trials is sparse.^9^ Understanding the cost of hospitalization is mandatory in building an investment case for accelerating vaccine development and implementation. This study will assist with understanding the cost of SAB among children and facilitate investment cases into pediatric vaccination, similar to those for group A and B *Streptococcus*, which have subsequently received substantial funding.^10^

There have been limited studies to date examining the cost of hospitalization due to pediatric SAB in Australia, making it difficult to understand the economic burden this disease places on the healthcare system. To bridge the knowledge gap and add to the evidence base, we aimed to investigate the cost of hospitalization in children with SAB.

## METHODOLOGY

We conducted an economic analysis involving children and young people ≤18 years old hospitalized between January 2017 and December 2018 with *S. aureus* identified from blood culture.

### Study Population

Patients were identified from the Invasive *Staphylococcus aureus* Infections and Hospitalisations (ISAIAH) cohort, a prospective multicenter cross-sectional study of pediatric SAB in Australia and New Zealand. Briefly, this was an observational study that collected demographic and clinical data from 552 individual patient episodes of SAB admitted to pediatric hospitals across Australia and New Zealand between 2017 and 2018.^2^ The ISAIAH cohort included 11 hospitals (8 tertiary and 3 secondary) across Australia and New Zealand (Table S1). Of these, 9 hospitals were located in Australia, spanning Western Australia, Victoria, New South Wales, South Australia, the Northern Territory, and Queensland. Collectively, these 9 Australian hospitals serve an estimated pediatric population of approximately 6 million children.^11^

### Setting and Location

Within the ISAIAH cohort, we explored the hospitalization cost of SAB among children admitted to Perth Children’s Hospital (PCH), formerly known as Princess Margaret Hospital. The hospital is the sole pediatric tertiary hospital in Western Australia (WA) and serves a pediatric population of approximately 700 000.^11^ WA occupies half of Australia’s landmass and comprises tropical, semiarid, and Mediterranean climate zones.

### Study Design

This observational, cross-sectional study did not involve any intervention or comparator group. Hospitalization costs were obtained from the PCH Business Intelligence Unit (BIU) for all patients with SAB identified from the ISAIAH cohort admitted to PCH.^2^ For each patient, costs comprised the direct costs of diagnosing and managing SAB, as well as a portion of the indirect costs of operating the tertiary hospital and providing healthcare, such as the cost of utilities, teaching and research, general cleaning and upkeep, maintenance, depreciation of the building and equipment, and other costs through external service providers.

These costs were provided following the National Hospital Cost Data Collection (NHCDC) specifications for Cost Centres and Line Items; each patient may have multiple Cost Centre records (eg, adolescent medicine, imaging, and pathology) for their hospitalization and each Cost Centre comprises multiple line-item costs (eg, nurse wages, allied health wages, consumables, and corporate costs).

To summarize these costs into broader, digestible categories, we aggregated the Cost Centre data into 8 Cost Centre groups following NHCDC specifications.^12^ These groups comprise (1) Clinical and (2) Allied Health service costs related largely to clinical and allied health services outside of those provided in emergency departments, operating rooms, and intensive care units; (3) Emergency Department; (4) Intensive Care; (5) Operating Room; (6) Pathology; (7) Imaging; and (8) Pharmacy. The distribution of NHCDC Line Items by Cost Centre group is summarized in Table S2. Definitions for each line item are provided in Table S3.

Despite the detailed level of hospitalization costs provided as line items for each patient, we could not discern costs attributable to diagnosing and managing SAB from costs for diagnosing and managing concurrent conditions during the patient’s episode of care. This is particularly relevant to patients with hospital-onset SAB. Therefore, we analyzed total hospitalization admission costs for the overall cohort and then focused on the costs of patients with community-onset SAB to facilitate the analysis of the direct admission costs associated with SAB rather than other comorbidities frequently implicated in hospital-onset SAB.

The primary outcome was the mean and total cost of community-onset SAB hospitalization for children admitted to PCH during the study period. As a secondary outcome, we calculated the national cost burden by extrapolating the mean admission cost for community-onset SAB from PCH to all ISAIAH participants with community-onset SAB across Australia. All the reported costs are in 2017-2018 Australian dollars (A$). As this was a cross-sectional study, we did not project or measure outcomes over an extended time horizon, and therefore, there were no future costs that required discounting.

### Extrapolation of Cost to the National ISAIAH Cohort

**Figure 1:**
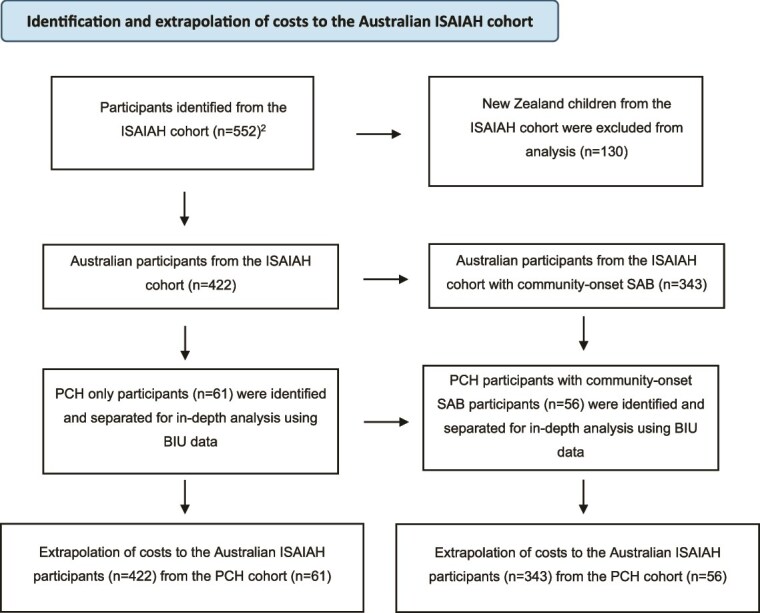
Identification and Extrapolation of Costs to the Australian ISAIAH Cohort

We estimated the cost of pediatric SAB hospitalization for the ISAIAH cohort of Australian patients by multiplying the mean cost per child hospitalized with SAB from the PCH cohort by the total number of children hospitalized with community-onset SAB within the Australian ISAIAH dataset (*n* = 343)^2^ over 2 years (Figure 1). The annual cost was then calculated by dividing the total cost by 2. Baseline characteristics between children admitted to PCH and those admitted to other pediatric Australian hospital study sites within the ISAIAH dataset were compared before extrapolation to determine any biases.

### Variables

Key demographic and clinical variables that were captured by the ISAIAH study were used to summarize the PCH cohort and analyze costs. These variables comprised transfer from a peripheral hospital, sex, Aboriginal and/or Torres Strait Islander status, antibiotic susceptibility profile, hospital or community-onset infection (hospital- and community-onset SAB was defined as a positive blood culture(s) collected >48 hours or ≤48 hours after presentation, respectively), focus of infection, comorbidities, length of stay (LOS), Socio-Economic Indexes for Areas (SEIFA), and the Accessibility/Remoteness Index of Australia (ARIA). The SEIFA quintiles (1 indicates the most disadvantaged areas and 5 indicates the most advantaged areas) and ARIA scores (classified into Major Cities, Inner Regional, Outer Regional, Remote, and Very Remote areas) are from national census data and were calculated using the individual’s residing postcode at the time of admission.^13^

### Analysis

We summarized demographic and clinical profiles using the median and interquartile range (IQR) for continuous variables and frequency for categorical variables. We compared the profiles between the ISAIAH PCH and non-PCH cohorts and, within the PCH cohort, between methicillin-susceptible *S. aureus* (MSSA) and methicillin-resistant *S. aureus* (MRSA) infections.

It is well established that healthcare costs do not follow a normal distribution, with a natural lower bound of 0 and often a few complex, high-cost patients. While the median and IQR are traditionally used to summarize skewed data, it is recommended that the mean be used to summarize and analyze healthcare costs because it reflects the entire dataset and facilitates exploration of the total cost in other cohorts.^14^ Therefore, we used the Shapiro–Wilk test to confirm that the patient-level cost distribution does not follow a normal distribution. Given a skewed distribution, we summarized and compared patient-level costs across key variables using the mean and 95% CI, which was calculated by the nonparametric bootstrap with 10 000 repetitions. A difference in mean costs was considered statistically significant when the 95% CIs did not overlap. All the statistical analyses were performed using STATA 18·0 (StataCorp LLC).

### Ethics Approval

Ethics approval was obtained from the Child and Adolescent Health Service of Western Australia Human Research Ethics Committee (2016161EP/RGS2587). We followed the Consolidated Health Economic Evaluation Reporting Standards 2022 (CHEERS 2022) Statement (Appendix A).

## RESULTS

### Baseline Characteristics

There were 56 patients with community-onset SAB admitted to PCH between January 2017 and December 2018 and included in the analyses (Table 1). Of those, 26 (46%) were female and 14 (25%) identified as Aboriginal and/or Torres Strait Islander. The median age was 7 years (IQR 2–11 years). MSSA bacteremia was the dominant antibiotic susceptibility phenotype (46/56 [82%]). Community onset was the most frequent SAB classification in the cohort (56/61 [92%]), and there was 1 death (2%) in the cohort; 27% (15/56) of the cohort had a skin (including eczema, furuncles, and skin trauma) related comorbidity (Table 1).

**Table 1:** Baseline Characteristics of SAB Patients from Perth Children’s Hospital and Other Australian Paediatric Hospitals

Baseline characteristics	Total (PCH) community-onset only	MSSA (PCH) community onset	MRSA (PCH) community onset	Total (other) community onset
**Pediatric SAB**	56	46 (82%)	10 (18%)	287
**(1) Demographics and setting**				
**Age (median)—years**	7 (IQR 2-11)	7 (IQR 3-11)	5 (IQR 1-9)	8 (IQR 2-11)
**Age groups**				
≤28 days, *n* (%)	1 (2)	1 (2)	0	15 (5)
>28 days to ≤1 years, *n* (%)	9 (16)	7 (15)	2 (20)	35 (12)
> 1 to ≤5 year, *n* (%)	11 (20)	8 (17)	3 (30)	61 (21)
≥ 5 to ≤18 years, *n* (%)	35 (62)	30 (65)	5 (50)	176 (61)
**Sex (Female)**	26 (46)	21 (46)	5 (50)	105 (37)
**Ethnicity (Aboriginal and/or Torres Strait Islander)**, *n* (%)	14 (25)	6 (13)	8 (80)	28 (10)
**Location**				
Transferred from a peripheral hospital, *n* (%)	22 (39)	15 (32)	7 (70)	87 (30)
**(2) Onset and epidemiological classification**				
**Classification**				
Hospital-onset	N/A	N/A	N/A	N/A
Community-onset				
**Classification**				
Healthcare-associated^a^, *n* (%)	10 (18)	9 (20)	1 (10)	52 (18)
Device, *n* (%)	7 (13)	6 (13)	1 (10)	41 (14)
Surgery, *n* (%)	2 (4)	2 (4)	0	7 (2)
Neutropenia, *n* (%)	4 (7)	4 (9)	0	13 (5)
**(3) Comorbidities**				
Hematological malignancy, *n* (%)	4/56 (7)	4/46 (9)	0	15 (5)
Solid organ malignancy, *n* (%)	2/56 (4)	2/46 (4)	0	18 (6)
Congenital heart disease, *n* (%)	5/56 (9)	5/46 (11)	0	10 (4)
Chronic renal disease, *n* (%)	1/56 (2)	1/46 (2)	0	4 (1)
Furuncles, *n* (%)	6/56 (10)	2 (4)	4 (40)	10 (3)
**(4) Predisposing conditions and exposures**				
Eczema, *n* (%)	4 (7)	4 (9)	0	26 (9)
Skin trauma, *n* (%)	5 (9)	1 (2)	4 (40)	6 (2)
Influenza, *n* (%)	1 (2)	1 (2)	0	10 (4)
Indwelling intravascular device present, *n* (%)	7 (13)	6 (13)	1 (10)	46 (16)
Recent surgery, *n* (%)	2 (4)	2 (4)	0	7 (2)
Neutropenia at onset, *n* (%)	4 (7)	4 (9)	0	13 (5)
**(5) Primary clinical focus of bacteremia**				
Skin and soft tissue infection (including furuncles), *n* (%)	8 (14)	7 (15)	1 (10)	48 (17)
Bone and joint infection, *n* (%)	37 (66)	30 (65)	7 (70)	165 (58)
Intravascular or implanted device-related infection, *n* (%)	7 (13)	6 (13)	1 (10)	46 (16)
Endocarditis, *n* (%)	5 (9)	5 (11)	0	13 (5)
Pneumonia, *n* (%)	7 (13)	3 (7)	4 (40)	32 (11)
Deep tissue, *n* (%)	17 (30)	12 (26)	5 (50)	73 (25)
No identified focus, *n* (%)	6 (11)	6 (13)	0	32 (11)

^a^Healthcare-associated is defined as device-related and/or surgery in the preceding 30 days of SAB and/or neutropenia < 1 × 10^9^/L).

Abbreviations: LOS, length of stay; MRSA, methicillin-resistant *Staphylococcus aureus*; MSSA, methicillin-susceptible *S. aureus*; PCH, Perth Children’s Hospital; SAB, *S. aureus* bacteremia.

Baseline characteristics were similar between participants in the PCH cohort compared with those from other pediatric sites across Australia (Table 1). However, the proportion of Aboriginal and/or Torres Strait Islander children was higher at PCH, at 25% (14/56), compared with 10% (28/287) at other Australian sites. The baseline characteristics between all PCH participants with SAB, including hospital-onset cases, and all SAB participants from other pediatric sites across Australia were compared and, similarly, baseline characteristics were similar, except the proportion of hospital-onset bacteremia was lower at PCH at 8% (5/61) compared with 21% (74/361) at other sites.

### Cost of Hospitalization within the PCH Cohort

The distribution of costs in the PCH cohort was positively skewed (Figure S1; *P* < .005). Approximately 60% of the cohort had a total hospitalization cost ≤A$40 000 (Figure S1). The mean cost of hospitalization per patient was A$53 037 [95% CI A$44 623-A$61 452] and the total cost across 56 patients over 24 months was A$2 970 115 (Table 2). The median length of hospitalization was 9 (IQR 6-15) days. General clinical services, with related indirect costs, comprised 73% of the total hospitalization cost, while 7% of the total cost was attributable to ICU services (Table 2).

**Table 2:** Summary of SAB Hospitalization Cost by National Hospital Cost Data Collection Cost Centre Groups (Community Onset Only)^a^

NHCDC Cost Centre group	*n*	Mean (95% CI) ($)	Total ($)
Emergency	50	1075 (939-1212)	53 758
Allied Health	50	1397 (1061-1734)	69 867
Pathology	51	1269 (684-1853)	64 715
Imaging	48	2897 (2344-3450)	139 065
Pharmacy	54	2339 (1601-3076)	126 291
Operating room	34	4403 (3510-5297)	149 731
Clinical	56	38 456 (31 786-45 126)	2 153 539
ICU	6	35 525 (21 650-49 399)	213 149
**Total**	**56**	**53 037 (44 623-61 452)**	**2 970 115**

^a^Not all patients had costs for all fields incurred at PCH. Abbreviations: NHCDC, National Hospital Cost Data Collection Cost Centre Groups; SAB, *Staphylococcus aureus* bacteremia; ICU, Intensive Care Unit.

The mean cost of MRSA bacteremia (median LOS [11 days (IQR 7-19)]) affecting 10 (18%) patients in our cohort was A$56 984[95% CI A$41 389-A$72 580], while the mean cost of MSSA bacteremia (median LOS [8 days (IQR 5-12)]) affecting 46 (82%) patients was A$52 180 [95% CI A$42 296-A$62 063], indicating no statistical difference in cost between the two groups (Table 3). Similarly, we did not find a statistically significant difference in the average cost between patients with MRSA bacteremia and those with MSSA bacteremia in the overall group (including hospital-onset) (Table S6).

**Table 3:** Summary of SAB Hospitalization Costs by Antibiotic Susceptibility Profiles for the PCH Cohort (Community Onset Only)^a^

NHCDC Cost Centre Group	MSSA			MRSA		
	*n*	Mean (95% CI) ($)	Total, $ (%)	*n*	Mean (95% CI) ($)	Total, $ (%)
Emergency	41	1092 (998-1187)	44 779 (2)	9	998 (865-1130)	8979 (2)
Allied Health	40	1141 (602-1680)	45 644 (2)	10	2422 (558-4287)	24 223 (4)
Pathology	41	1267 (697-1837)	51 930 (2)	10	1279 (717-1840)	12 786 (2)
Imaging	39	2823 (1645-4001)	110 087 (5)	9	3220 (1680-4760)	28 978 (5)
Pharmacy	43	2214 (777-3651)	95 205 (4)	10	3109 (507-5711)	31 086 (5)
Operating room (	26	4552 (3187-5917)	118 351 (5)	8	3922 (2536-5309)	31 380 (5)
ICU	5	19 959 (3264-36 653)	99 793 (4)	1	113 355 (na)	113 355 (20)
Clinical	46	39 848 (24 353-55 343)	1 833 010 (76)	10	32 053 (25 894-38 212)	320 529 (56)
Total	46	52 180 (42 296-62 063)	2 398 799	10	56 984 (41 389-72 580)	517 316

^a^Not all patients had costs for all fields incurred at PCH. Abbreviations: ICU, intensive care unit; MRSA, methicillin-resistant *Staphylococcus aureus*; MSSA, methicillin-susceptible *S. aureus*; PCH, Perth Children’s Hospital; SAB, *Staphylococcus aureus* bacteremia.

Table 4 reports the cost of community-onset SAB hospitalization across a range of key covariates (*n* = 56). Mean costs were much higher for patients who had a healthcare-associated infection, LOS of more than 30 days, and a multifocal infection (>1 site involved; Table 4). Higher costs were also observed in those with a primary clinical focus of skin and soft tissue infection (including furuncles), intravascular or implanted device–related infection, and endocarditis (Table 4).

**Table 4:** Summary of SAB Hospitalization Cost by Key Covariates for the PCH Cohort (Community Onset Only, *n* = 56)

Variable	*n* (%)	Mean (95% CI) ($)
**Age groups**		
0-2 years	14 (25)	54 551 (27 580-81 521)
3-5 years	8 (14)	30 570 (19 112-42 027)
6-8 years	12 (21)	22 146 (9610-34 681)
9-11 years	10 (18)	83 992 (20 559-147 425)
12-14 years	7 (13)	59 731 (5290-114 173)
15-17 years	5 (9)	87 613 (31 157-144 069)
**Sex**		
Male	30 (54)	55 390 (33 736-77 045)
Female	26 (46)	50 323 (22 598-78 048)
**Ethnicity**		
Aboriginal and/or Torres Strait Islander	14 (25)	59 212 (34 609-83 815)
Other	42 (75)	50 979 (29 418-72 541)
**Susceptibility profiles**		
MSSA	46 (82)	52 148 (32 176-72 119)
MRSA	10 (18)	57 132 (25 801-88 463)
**Healthcare-associated**		
Yes	10 (18)	94 781 (37 712-151 850)
No	46 (82)	43 963 (28 460-59 466)
**ARIA**		
Inner Regional	11 (20)	45 774 (4 957-96 505)
Major City	25 (45)	58 227 (35 016-81 438)
Outer Regional	5 (9)	30 533 (19 908-41 159)
Remote	9 (16)	56 054 (17 729-94 379)
Very Remote	6 (11)	58 962 (10 724-107 200)
**SEIFA quintiles**		
1 (most disadvantaged)	11 (20)	43 138 (15 385-70 891)
2	15 (27)	74 478 (30 350-118 607)
3	12 (21)	54 486 (17 340-91 632)
4	11 (20)	49 005 (12 468-85 543)
5 (most advantaged)	7 (13)	26 505 (22 986-30 024)
**LOS**		
Less than 30 days	47 (84)	31 450 (23 078-39 822)
30 days or more	9 (16)	165 776 (111 599-219 953)
**Comorbidities (*n*)**		
0	27 (48)	35 809 (18 660-52 959)
1	21 (38)	38 743 (24 581-52 905)
2	7 (13)	166 557 (100 815-232 298)
>3	1 (2)	23 761 (NA)
**ID consult**		
No	17 (30)	21 169 (10 887 - 31 450)
Yes	39 (70)	66 929 (43 932 -89 927)
**Multifocal disease (>1 site involved)**		
No	45 (80)	38 774 (25 405 -52 143)
Yes	11 (20)	111 391 (54 431-168 350)
**Primary clinical focus of bacteremia**		
Skin and soft tissue infection (including furuncles)	8 (14)	106 435 (18 197-194 672)
Bone and joint infection	37 (66)	44 803 (29 067 – 60 539)
Intravascular or implanted device-related infection	7 (13)	132 554 (56 646-208 462)
Endocarditis	5 (9)	215 582 (165 198-265 965)
Pneumonia	7 (13)	93 830 (43 742-143 918)
Deep tissue	17 (30)	66 589 (37 711-95 467)
No identified focus	6 (11)	21 764 (16 627-26 901)

Abbreviations: ARIA, Accessibility/Remoteness Index of Australia; ID, Infectious Diseases; LOS, length of stay; PCH, Perth Children’s Hospital; SAB, *Staphylococcus aureus* bacteremia; SEIFA, Socio-Economic Indexes for Areas.

### Cost of Hospitalization Stratified by Classification of SAB

The overall total cost of SAB hospitalization among the 61 patients with both hospital-onset and community-onset SAB was A$4 264 377, with a mean cost of A$69 908 [95% CI A$42 572-A$97 243] (Table S5). The total cost of SAB hospitalization among the 5 (8%) patients with hospital-onset infections was A$1 294 259, with a mean cost of A$258 852 [95% CI A$35 003-A$482 700] (Table S7).

### Total Cost of Hospitalization (ISAIAH Cohort Only)

Extrapolating across 343 Australian pediatric community-onset SAB patients in the ISAIAH dataset from 2017 to 2018, the estimated total cost of hospitalization was A$18 185 691 [95% CI A$15 305 689-A$21 078 036]. The estimated annual total cost of hospitalization across SAB pediatric patients in Australia within ISAIAH was A$9 092 846 [95% CI A$7 652 845-A$10 539 018].

## DISCUSSION

This study reports, for the first time, the hospitalization costs for pediatric SAB. Our key findings include hospitalization costs for pediatric community-onset SAB exceed A$9 million per year across Australia and there was no difference in hospitalization costs by antibiotic susceptibility profile. These findings add to the current understanding of the economic burden of community-onset pediatric SAB within Australia and underscore the need for effective prevention strategies.

The overall average costs of community-onset SAB hospitalization in children reported here ($53 037 [95% CI A$44 623-A$61 452) are comparable to the costs reported in other key-priority, high-frequency sepsis pathogens such as group A *Streptococcus*^15^ and all-cause pediatric sepsis hospitalizations from Australia and New Zealand.^16^ A recent Australian pediatric study (*n* = 65) reported a mean cost of A$60 112 (SD A$79 072.41) for invasive group A *Streptococcal* (iGAS) hospitalizations in children.^15^ ICU admission was the largest cost driver in the iGAS cohort;^15^ however, the average cost of ICU admission in childhood *S. aureus* sepsis in our study was approximately 1.5 times higher than iGAS (A$35 525; *n* = 6 vs A$23 156; *n* = 27) despite a substantially lower ICU admission rate, reflecting the high complexities and length of stay of SAB cases requiring critical care. Additionally, a study from Australia and New Zealand revealed that the mean sepsis and septic shock hospitalization costs in children were A$62 062 per patient.^16^ While the average cost in that study was higher than the cost reported in our SAB cohort, our main analysis was restricted to community-onset SAB cases to reduce confounding from complex hospital-onset SAB. As such, our cost estimates may underestimate the true economic burden of pediatric SAB.

Our study found no significant difference in hospitalization costs between MSSA and MRSA bacteremia cases. While previous literature has predominantly focused on the costs and healthcare burden of MRSA infections in children and adults,^17^ our findings suggest that the economic impact of MSSA is equally high and should not be overlooked. Large international studies, including those from Japan and Australia, have reported higher hospitalization costs for MRSA bacteremia in adults.^18^^,^^19^ However, two large US studies of over 50 000 SAB cases have found minimal or no difference in costs between MRSA versus MSSA bacteraemia.^20^^,^^21^ including near-identical admission costs (A$35 623 vs A$35 369)^20^ and another showing no significant difference (A$51 789 vs A$51 263; *P* = .69).^21^ This variability in costs by resistance phenotype may reflect differences in population characteristics, healthcare settings, and evolving *S. aureus* virulence in Australia and Japan but has not been consistent across the world in large studies. As such, these studies are not direct comparators to our findings but rather serve to demonstrate the broad spectrum of the disease severity and associated health utilization patterns across different contexts and populations. Furthermore, the limited availability of pediatric-specific SAB costing data, particularly studies encompassing both MSSA and MRSA, highlights the need for further research to validate these findings to inform cost-effectiveness analysis and prioritize sepsis prevention strategies.

Given the absence of a licensed vaccine for *S. aureus*, the implementation of cost-effective prevention measures to reduce hospital admissions is imperative, as over 90% of cases were community-onset, and 27% of children in the PCH cohort with community-onset SAB presented with skin-related comorbidities, such as skin trauma, furuncles, and eczema. The heavy burden of skin infections in Aboriginal and/or Torres Strait Islander children in Australia is well described and is higher than in any other population globally.^22–24^ Aboriginal and/or Torres Strait Islander children constituted 25% of the PCH cohort despite comprising only 3.3% of the state population.^25^ The disproportionately high burden of bacterial skin infections among Aboriginal and/or Torres Strait Islander children highlights the importance of developing comprehensive guidelines for skin health^26^ and implementing targeted skin infection control programs.^27^ Such interventions have the potential to substantially reduce the incidence of bacterial skin infections and, consequently, SAB in vulnerable populations. Similarly, hospital-based infection prevention and control programs, including hand hygiene, aseptic technique, and isolation precautions, as well as *S. aureus* decolonization prior to surgery or device insertion, should remain a priority, as 24% of our cohort developed a healthcare-associated infection (Table S4) and hospital-onset SAB (Table S7) was significantly more costly than community-onset SAB.

### Limitations

Our study has several limitations. First, a relatively small number of community-onset SAB hospitalizations during the study period limited the statistical power to detect differences in mean costs between subgroups, particularly within the MRSA subgroup. Larger multisite studies in childhood SAB are required to strengthen the evidence for where length of stay and surgical interventions may result in cost differences between MSSA and MRSA. Second, we were unable to separate costs attributable to diagnosing and managing SAB from costs associated with diagnosing and managing concurrent conditions during the patient’s episode of care for hospital-onset infection. However, we mitigated this by analyzing patients admitted with community-onset SAB separately to hospital-onset SAB. Furthermore, we did not estimate the cost of SAB from a societal or family perspective by including missed days of work and school during hospital stay, thus underestimating the true cost of SAB. This study was performed in Australia, a high-income country with free universal healthcare access, and the majority of the cases within the PCH cohort were community-onset patients. The generalizability of the costs to other countries will be affected by several factors, including wage rates, access to highly specialized clinical and diagnostic services, prevalence of patient comorbidities, funding mechanisms such as private insurance, and other capital and infrastructure costs. However, the underlying frequency of resource use, such as length of stay, may be useful for other settings. Our study reports cases with respective costing data from 2017 to 2018, and inflation is likely to place ongoing upward pressure on these costs.

## CONCLUSION

A comprehensive costing of SAB in children is essential for understanding its economic burden on the healthcare system. Our study provides a foundation for future cost-effectiveness analyses to assess the cost implications of novel interventions and prevention strategies. We found that the average cost of community-onset SAB hospitalization in children from our cohort was A$53 000, with an extrapolated annual estimate of A$9 million across the complete Australian ISAIAH dataset. Our data suggest the economic burden of childhood SAB is substantial, irrespective of the antibiotic susceptibility profile. Prevention and treatment efforts should remain focused on *S. aureus* overall, including both skin control programs in the community and infection prevention in the hospital.

## Supplementary Material

Supporting_information_01122025_piaf114

CHEERS_Checklist_Appendix_A_piaf114

## Data Availability

The data for this study will not be shared, as we do not have permission from the participants or ethics approval to do so.

## References

[1] Campbell AJ, Daley DA, Bell JM et al. Progress towards a coordinated, national paediatric antimicrobial resistance surveillance programme: *Staphylococcus aureus*, enterococcal and Gram-negative bacteraemia in Australia. *J Antimicrob Chemother* 2020;75:1639–1644. 10.1093/jac/dkaa065 32155261

[2] Campbell AJ, Al Yazidi LS, Phuong LK et al. Paediatric *Staphylococcus aureus* bacteraemia: clinical spectrum and predictors of poor outcome. *Clin Infect Dis* 2022;74:604–613. 10.1093/cid/ciab510 34089594

[3] World Health Organization . WHO Bacterial Priority Pathogens List, 2024. Geneva: WHO, 2024.

[4] Anpalagan K, Dotel R, MacFadden DR et al. Does adjunctive clindamycin have a role in *Staphylococcus aureus* bacteraemia? A protocol for the adjunctive treatment domain of the Staphylococcus aureus Network Adaptive Platform (SNAP) randomised controlled trial. *Clin Infect Dis* 2024;79:626–634. 10.1093/cid/ciae289 38801783 PMC11426255

[5] de Kretser D, Mora J, Bloomfield M et al. Early oral antibiotic switch in *Staphylococcus aureus* bacteraemia: the SNAP trial early oral switch protocol. *Clin Infect Dis* 2024;79:871–887. 10.1093/cid/ciad666 37921609 PMC11478773

[6] Tong SYC, Mora J, Bowen AC et al. The SNAP trial protocol: new tools for an old foe. *Clin Infect Dis* 2022;75:2027–2034. 10.1093/cid/ciac476 35717634 PMC9710697

[7] Campbell AJ, Anpalagan K, Best EJ et al. Whole-of-life inclusion in Bayesian adaptive platform clinical trials. *JAMA Pediatr* 2024;178:1066–1071. 10.1001/jamapediatrics.2024.2697 39158898

[8] Anpalagan K, Bowen AC, Lamborn L et al. Optimising detection of thrombosis in paediatric *Staphylococcus aureus* bacteraemia: a prospective interventional sub-study protocol. *Infect Dis Now* 2025;55:105010. 10.1016/j.idnow.2024.105010 39542423

[9] Frost I, Sati H, Garcia-Vello P et al. The role of bacterial vaccines in the fight against antimicrobial resistance: an analysis of the preclinical and clinical development pipeline. *Lancet Microbe* 2023;4:e113–e125. 10.1016/S2666-5247(22)00303-2 36528040 PMC9892012

[10] Cannon JW, Jack S, Wu Y et al. An economic case for a vaccine to prevent group A *Streptococcus* skin infections. *Vaccine* 2018;36:6968–6978. 10.1016/j.vaccine.2018.10.001 30340879

[11] Australian Bureau of Statistics . Quarterly Population Estimates (ERP), by State/Territory, Sex and Age, 2024–Q2. Canberra: ABS; 2024. Accessed 15 January 2025. https://dataexplorer.abs.gov.au/

[12] Independent Hospital Pricing Authority . National Hospital Cost Data Collection: Private Hospital Report Round 2023 (Financial Year 2018–19). Sydney: IHPA, 2021.

[13] Australian Bureau of Statistics . Construction of the Indexes. Canberra: ABS; 2021. Accessed 10 February 2025. https://www.abs.gov.au/statistics/detailed-methodology-information/concepts-sources-methods/socio-economic-indexes-areas-seifa-technical-paper/2021/construction-indexes

[14] Thompson SG, Barber JA. How should cost data in pragmatic randomised trials be analysed? *BMJ* 2000;320:1197–1200. 10.1136/bmj.320.7243.1197 10784550 PMC1127588

[15] Brusco NK, Oliver J, McMinn A, Steer A, Crawford N. The cost of care for children hospitalised with invasive Group A *Streptococcus* disease in Australia. *BMC Health Serv Res* 2021;21:1340. 10.1186/s12913-021-07265-8 34906126 PMC8670128

[16] Schlapbach LJ, Straney LDJ, Alexander J et al. Mortality related to invasive infections, sepsis, and septic shock in critically ill children in Australia and New Zealand, 2002–13: a multicentre retrospective cohort study. *Lancet Infect Dis* 2015;15:46–54. 10.1016/S1473-3099(14)71003-5 25471555

[17] Lee BY, Singh A, David MZ et al. The economic burden of community-associated methicillin-resistant *Staphylococcus aureus* (CA-MRSA). *Clin Microbiol Infect* 2013;19:528–536. 10.1111/j.1469-0691.2012.03914.x 22712729 PMC3463640

[18] Shoji T, Muto R, Fukuda H, Muraki Y, Kawata K, Akazawa M. Cost and healthcare utilisation of methicillin-resistant *Staphylococcus aureus* bacteraemia estimated from linked antimicrobial resistance surveillance and hospital claims data in Japan. *Antimicrob Steward Healthc Epidemiol* 2022;2:e147. 10.1017/ash.2022.280 36483379 PMC9726553

[19] Miyakis S, Brentnall S, Masso M et al. Key predictors and burden of meticillin-resistant *Staphylococcus aureus* infection compared with meticillin-susceptible *S aureus* infection in an Australian hospital setting. *J Hosp Infect* 2022;129:41–48. 10.1016/j.jhin.2022.07.004 35839999

[20] Inagaki K, Lucar J, Blackshear C, Hobbs CV. Methicillin-susceptible and methicillin-resistant *Staphylococcus aureus* bacteraemia: nationwide estimates of 30-day readmission, in-hospital mortality, length of stay, and cost in the USA. *Clin Infect Dis* 2019;69:2112–2118. 10.1093/cid/ciz123 30753447

[21] Klein EY, Jiang W, Mojica N et al. National costs associated with methicillin-susceptible and methicillin-resistant *Staphylococcus aureus* hospitalisations in the USA, 2010–14. *Clin Infect Dis* 2019;68:22–28. 10.1093/cid/ciy399 29762662 PMC6293004

[22] Ricciardo BM, Kessaris HL, Nannup N et al. Skin health of Aboriginal children living in urban communities. *Australas J Dermatol* 2024;65:e224–e237. 10.1111/ajd.14363 39205508 PMC11629136

[23] Bowen AC, Mahé A, Hay RJ et al. The global epidemiology of impetigo: a systematic review of the population prevalence of impetigo and pyoderma. *PLoS One* 2015;10:e0136789. 10.1371/journal.pone.0136789 26317533 PMC4552802

[24] Romani L, Steer AC, Whitfeld MJ, Kaldor JM. Prevalence of scabies and impetigo worldwide: a systematic review. *Lancet Infect Dis* 2015;15:960–967. 10.1016/S1473-3099(15)00132-2 26088526

[25] Australian Bureau of Statistics . Western Australia: Aboriginal and Torres Strait Islander Population Summary. Canberra: ABS; 2022. Accessed 15 January 2025. https://www.abs.gov.au/articles/western-australia-aboriginal-and-torres-strait-islander-population-summary

[26] The Australian Healthy Skin Consortium . National Healthy Skin Guideline: For the Diagnosis, Treatment and Prevention of Skin Infections for Aboriginal and Torres Strait Islander Children and Communities in Australia (2nd edn). 2023.

[27] Thomas HMM, Enkel SL, Mullane M et al. Trimodal skin health programme for childhood impetigo control in remote Western Australia (SToP): a cluster randomised, stepped-wedge trial. *Lancet Child Adolesc Health* 2024;8:809–820. 10.1016/S2352-4642(24)00229-3 39393383

