# Supplementary material for: The Hospitalization Cost of Pediatric Staphylococcus aureus Bacteremia
Source: J Pediatric Infect Dis Soc. 2025 Dec 19;15(1):piaf114. doi: 10.1093/jpids/piaf114 (PMC12821364; doi:10.1093/jpids/piaf114)
Supplement: Supporting_information_01122025_piaf114 [file supporting_information_01122025_piaf114.docx]

Table 1. List of hospitals included in the ISAIAH Study

| **Hospital** | **Location** |
| --- | --- |
| Perth Children’s Hospital (PCH) | Perth, Western Australia |
| Royal Children’s Hospital (RCH) | Melbourne, Victoria |
| Women’s and Children’s Hospital (WCH) | Adelaide, South Australia |
| Sydney Children’s Hospital (SCH) Randwick | Sydney, New South Wales |
| The Children’s Hospital at Westmead (CHW) | Sydney, New South Wales |
| Monash Children’s Hospital | Melbourne, Victoria |
| Queensland Children’s Hospital (QCH) | Brisbane, Queensland |
| Royal Darwin Hospital | Darwin, Northern Territory |
| Starship Children’s Hospital | Auckland, New Zealand |
| Middlemore Hospital (Kidz First) | Auckland, New Zealand |
| University Hospital Geelong | Geelong, Victoria |

Table 2. Corresponding NHCDC line items to NHCDC Cost Centre Groups

| **Sum of Total Cost ($)** | CORP | DeprecB | DeprecE | GS | Hotel | IMAG | Lease | MS | OnCosts | Path | PatTrav | PharmNPBS | PharmPBS | Pros | PROSTH | RESEARCH | SWAH | SWMed | SWNurs | SWOther | SWVMO | TEACHING | Grand Total |
| --- | --- | --- | --- | --- | --- | --- | --- | --- | --- | --- | --- | --- | --- | --- | --- | --- | --- | --- | --- | --- | --- | --- | --- |
| Allied | 7% | 0% | 1% | 6% | 2% | 0% | 0% | 3% | 7% | 0% | 0% | 0% | 1% | 0% | 0% | 1% | 57% | 0% | 1% | 12% | 0% | 2% | 100% |
| Clinical | 5% | 0% | 2% | 6% | 5% | 0% | 0% | 4% | 7% | 0% | 0% | 0% | 0% | 0% | 0% | 1% | 6% | 22% | 26% | 11% | 0% | 5% | 100% |
| Critical | 5% | 0% | 2% | 4% | 4% | 0% | 0% | 3% | 7% | 0% | 0% | 0% | 1% | 0% | 0% | 1% | 1% | 22% | 34% | 8% | 0% | 6% | 100% |
| Emergency | 5% | 0% | 1% | 4% | 3% | 0% | 0% | 3% | 7% | 0% | 0% | 0% | 0% | 0% | 0% | 2% | 1% | 35% | 23% | 10% | 0% | 6% | 100% |
| Imaging | 5% | 0% | 8% | 8% | 2% | 1% | 0% | 2% | 6% | 0% | 0% | 0% | 0% | 0% | 0% | 1% | 21% | 17% | 4% | 10% | 13% | 3% | 100% |
| Operating Room | 4% | 0% | 5% | 5% | 6% | 0% | 0% | 9% | 5% | 1% | 0% | 0% | 0% | 0% | 0% | 1% | 5% | 34% | 15% | 5% | 0% | 4% | 100% |
| Pathology | 23% | 0% | 0% | 3% | 0% | 0% | 0% | 0% | 0% | 70% | 0% | 0% | 0% | 0% | 0% | 0% | 0% | 0% | 0% | 3% | 0% | 0% | 100% |
| Pharmacy | 2% | 0% | 1% | 2% | 1% | 0% | 0% | 0% | 2% | 0% | 0% | 49% | 20% | 0% | 0% | 0% | 17% | 0% | 0% | 4% | 0% | 1% | 100% |
| Other | 4% | 0% | 0% | 4% | 0% | 0% | 0% | 0% | 1% | 0% | 0% | 0% | 0% | 71% | 16% | 0% | 0% | 0% | 0% | 3% | 0% | 0% | 100% |
| **Grand Total** | **6%** | **0%** | **2%** | **5%** | **4%** | **0%** | **0%** | **3%** | **6%** | **2%** | **0%** | **2%** | **1%** | **0%** | **0%** | **1%** | **7%** | **20%** | **23%** | **9%** | **0%** | **5%** | **100%** |

Abbreviation: National Hospital Cost Data Collection, NHCDC

Table 3. NHCDC Line-item definitions

| SWNurs | Nursing Salaries and Wages, including: • Registered Nurses;  • Enrolled Nurses; • Establishment Based Student Nurses; and • Trainee/pupil nurses. |
| --- | --- |
| SWMed | Medical Salary and Wages, including: • Specialist and General Practice Medical Officers; • Registrars;  • Residents; and • Interns. |
| SWVMO | Visiting Medical Officers (VMO) Salary and Wages |
| SWAH | Allied Health Salary and Wages, including: • Aboriginal and Torres Strait Islander health worker • Audiology • Chiropractic • Dietetics • Exercise physiology • Occupational therapy • Optometry • Oral health • Orthoptics • Orthotics and prosthetics • Osteopathy • Paramedicine • Physiotherapy • Podiatry • Psychology • Social work • Speech pathology |
| SWOther | Other staff, including: • Other Personal Care staff - Other Personal Care staff primarily provide personal care to patients or residents. These staff, however, are not formally qualified, and may be undergoing training in nursing or allied health professions. The function provided by these staff must not be an overhead in nature. Examples of staff in this category include: o attendants,  o assistants or home assistants,  o home companions,  o family aides,  o ward helpers, assistants or assistants in nursing • Other Administrative, Maintenance and Clerical Staff - staff engaged in administrative, maintenance and clerical duties. Staff in this category do not carry out services that are carried out by medical, nursing, diagnostic or health professionals. Examples of relevant staff include  o ward clerks; o health information managers and  administrative staff. |
| OnCosts | Labour (staff) oncosts, all staff types |
| Path | Goods and services used in the provision of a pathology service and consumables (including reagents, stains and calibration products, etc.) or the actual cost as billed by a provider. This includes the cost of pathology staff. |
| Imag | Imaging: Goods and services used in the provision of an imaging service (including film, contrast, etc.) or the actual cost as billed by a provider. This includes the cost of imaging staff. |
| Pros | Prostheses: Goods and services used in the provision of services to implant prostheses, human tissue item and other medical devices that are: specified on the Prostheses List; or  assessed as being comparable in function to devices on the Prostheses List. The Prostheses List is available at: http://www.health.gov.au/internet/main/publishing.nsf/content/health-privatehealth-prostheseslist.htm |
| MS | Medical and surgical supplies costs are goods and services used in the provision of, or subsequent treatment resulting from, surgical services excluding those used for prostheses and drugs. This could include treatments resulting from surgery, such as surgical wounds that require later attention, or bed sores resulting from a surgical episode. All other medical and surgical supplies. |
| GS | All other Goods and Services not else where described. |
| PharmPBS | Pharmacy PBS: Goods and services used in the provision of a pharmaceutical service and consumables or the actual cost as billed by a provider. They include the purchase, production, distribution, supply and storage of drug products and clinical pharmacy services of PBS-reimbursed pharmaceuticals. This includes the cost of pharmacy staff. |
| PharmNPBS | Non PBS pharmacy: Goods and services used in the provision of a pharmaceutical service and consumables or the actual cost as billed by a provider. This includes the purchase, production, distribution, supply and storage of drug products and clinical pharmacy services of PBS non-reimbursed pharmaceuticals. |
| Blood | Blood Products: Blood Products and Services are as defined under the National Blood Agreement. The National Blood Agreement is available at: www.blood.gov.au/national-blood-agreement |
| DeprecB | Building Depreciation: Building depreciation includes fixed fit-out such as items fitted to the building such as lights and partitions. |
| DeprecE | Equipment Depreciation: Equipment depreciation includes non-fixed building fit-out such as theatre tables, moveable furniture, and chemotherapy chairs. |
| Hotel | Hotel includes: • cleaning products and services; • linen and laundry services; • food services (patients); and  • general hotel services. This includes the cost of hotel staff. |
| Corp | Corporate costs (from outside the hospital GL and not otherwise specified)  The AHPCS V4.0 standard specifies that corporate costs should be mapped to goods and services. For R23 IHPA will accept line item classified as Corporate as a transitional arrangement. |
| Lease | Leasing costs: This category includes all operating leases in line with Australian Accounting Standards. Capital leases are excluded from this category. |
| Cap | Capital works - not in scope |
| Exclude | Excluded costs – not in scope |
| PatTrav | Patient travel: This category includes both emergency and non-emergency travel which contributes to an organisation’s day-to-day production of final products. |

Abbreviation: National Hospital Cost Data Collection, NHCDC

Table 4. Baseline characteristics of SAB patients from Perth Children’s Hospital and other Australian paediatric hospitals

|  |  |  |  |  |
| --- | --- | --- | --- | --- |
| **Baseline Characteristics** | **MSSA (PCH)** | **MRSA (PCH)** | **Total (PCH)** | **Total (other)** |
| **Paediatric SAB** | 50 (82%) | 11 (18%) | 61 | 361 |
| **Age (median) - years** | 7 (IQR 2-12) | 5 (IQR 1 – 9) | 7 (IQR 2 – 13) | 6 (IQR 1-11) |
| **Female** | 22 (44%) | 6 (55%) | 28 (46%) | 137 (38%) |
| **Aboriginal and/or Torres Strait Islander** | 7 (14%) | 9 (82%) | 16 (26%) | 35 (10%) |
| **Location** |  |  |  |  |
| Transferred from a peripheral hospital | 17 (34%) | 7 (64%) | 24 (39%) | 106 (29%) |
| **LOS (median) - days** | 10 (IQR 8-24) | 18 (IQR 11- 31) | 11 (IQR 9-27) | 15 (IQR 9 - 29) |
| **Classification** |  |  |  |  |
| Hospital-onset | 4 (8%) | 1 (9%) | 5 (8%) | 74 (21%) |
| Community-onset | 46 (92%) | 10 (91%) | 56 (92%) | 287 (80%) |
| **Classification**  *Healthcare-associated | 12 (24%) | 2 (18%) | 14 (23%) | 103 (29%) |
| *Device* | 9 (18%) | 2 (18%) | 11 (18%) | 83 (23%) |
| *Surgery* | 2 (4%) | 0 (0%) | 2 (3%) | 23 (6%) |
| *Neutropenia* | 5 (10%) | 1 (9%) | 6 (10%) | 17 (5%) |
| **Comorbidities**  Haematological malignancy  Solid organ malignancy  Congenital heart disease  Eczema  Chronic renal disease  Furuncles  Skin trauma  Influenza | 6 (12%)  2 (4%)  6 (12%)  4 (8%)  2 (4%)  2 (4%)  1 (2%)  1 (2%) | 1 (9%)  0  0  0  0  4 (36%)  4 (36%)  0 | 7/61(12%)  2/61 (3%)  6/61 (10%)  4/61 (7%)  2/61 (3%)  6/61 (10%)  5/61(8%)  1/61 (2%) | 22 (6%)  23 (6%)  28 /360 (8%)  29/361 (8%)  4/360 (1%)  11/361 (3%)  6/361 (2%)  11/360 (3%) |

Table 5. Summary of SAB Hospitalisation Cost by National Hospital Cost Data Collection Cost Centre Groups

| NHCDC Cost Centre Group | N | Mean (95% CI) | Total |
| --- | --- | --- | --- |
| Emergency | 52 | $1,072 ($994 - $1,150) | $55,742 |
| Allied Health | 55 | $1,945 ($1,109 - $2,781) | $106,998 |
| Pathology | 56 | $2,715 ($766 - $4,665) | $152,067 |
| Imaging | 53 | $3,450 ($2,277 - $4,624) | $182,875 |
| Pharmacy | 58 | $4,150 ($1,667- $6,633) | $240,689 |
| Operating room | 37 | $5,471 ($3,719 - $7,223) | $202,438 |
| Clinical | 61 | $41,086 ($28,288 - $53,884) | $2,506,230 |
| ICU | 7 | $116,763 ($37,551- 271,077) | $817,340 |
| Total | **61** | **$69,908 ($42,572 - $97,243)** | **$4,264,377** |

Abbreviations: National Hospital Cost Data Collection Cost Centre Groups, NHCDC; *Staphylococcus aureus* bacteraemia, SAB.

Note. Not all patients had costs for all fields incurred at PCH

Table 6. Summary of SAB Hospitalisation cost by National Hospital Cost Data Collection Cost Centre Groups and SAB susceptibility profiles for the PCH cohort

| **NHCDC Cost Centre Group** | **MSSA** | | | **MRSA** | | |
| --- | --- | --- | --- | --- | --- | --- |
|  | **N** | **Mean (95% CI)** | **Total (%)** | **N** | **Mean (95% CI)** | **Total (%)** |
| Emergency | 42 | $1,086 ($993 - $1,179) | $45,614 (1%) | 10 | $1,012 ($888 - $1,138) | $10,128 (1%) |
| Allied Health | 44 | $1,595 ($815 - $2,375) | $70,169 (2%) | 11 | $2,590 ($104 - $5,077) | $28,492 (3%) |
| Pathology | 45 | $2,746 ($306 - $5,186) | $123,574 (4%) | 11 | $3,348 ($898 - $5,798) | $36,828 (4%) |
| Imaging | 43 | $3,182 ($1,849 - $4,516) | $136,840 (4%) | 10 | $4,603 ($1,507 - $7,699) | $46,034 (6%) |
| Pharmacy | 47 | $3,247 ($1,283 - $5,210) | $152,588 (4%) | 9 | $5,510 ($2,440 - $8,581) | $49,592 (6%) |
| Operating room | 28 | $5,459 ($3,486 - $7,432) | $152,845 (4%) | 11 | $8,009 ($1,272- $17,290) | $88,100 (11%) |
| Clinical | 50 | $40,969 ($26,364 - $55,573) | $2,048,426 (60%) | 11 | $41,618 ($23,321- $59,915) | $457,803 (55%) |
| ICU | 6 | $117,331 ($58,998- $293,659) | $703,985 (21%) | 1 | $113,355 (NA) | $113,355 (14%) |
| **Total** | **50** | **$ 68,681 ($36,313 - $101,048)** | **$3,434,043 (100%)** | **11** | **$75,485 ($32,770 - $118,199)** | **$830,334 (100%)** |

Abbreviations: Perth Children’s Hospital, PCH; *Staphylococcus aureus* bacteraemia, SAB; Methicillin susceptible *Staphylococcus aureus*, MSSA; Methicillin-resistant *Staphylococcus aureus*, MRSA. Note. Not all patients had costs for all fields incurred at PCH

Table 7. Summary of SAB Hospitalisation cost by key covariates for the PCH cohort (n=61)

| **Variable** | **N (%)** | | **Mean (95% CI)** |
| --- | --- | --- | --- |
| **Age groups** | | | |
| 0-2 years | 16 (26%) | $98,892 ($15,826- $181,957) | |
| 3-5 years | 9 (15%) | $55,953 ($8,082 - $103,823) | |
| 6-8 years | 12 (20%) | $22,146 ($8,394 - $35,897) | |
| 9-11 years | 10 (16%) | $83,992 ($21,787 - $146,197) | |
| 12-14 years | 8 (13%) | $73,829 ($21,665 - $125,992) | |
| 15-17 years | 6 (10%) | $80,372 ($28,409 - $132,335) | |
| **Sex** |  |  |  |
| Male | 33 (54%) | $76,498 ($30,596- $122,400) | |
| Female | 28 (46%) | $62,140 ($32,178 -$92,103) | |
| **Ethnicity** |  |  |  |
| Aboriginal and/or Torres Strait Islander | 16 (26%) | $70,760 ($38,625 - $102,894) | |
| Other | 45 (74%) | $69,605 ($32,686- $106,523) | |
| **Classification** |  |  |  |
| Community-onset | 56 (92%) | $53,038 ($36,042 -$70,033) | |
| Hospital-onset | 5 (8%) | $258,852 ($35,003- $482,700) | |
| **ARIA** |  |  |  |
| Inner Regional | 11 (18%) | $45,774 ($6,713 - $98,261) | |
| Major City | 29 (47%) | $92,122 ($39,545 - $144,699) | |
| Outer Regional | 6 (10%) | $38,511 ($20,957- $56,067) | |
| Remote | 9 (15%) | $56,054 ($14,706 - $97,402) | |
| Very Remote | 6 (10%) | $58,962 ($9,761 - $10,8163) | |
| **SEIFA Quintiles** | | | |
| 1 (most disadvantaged) | 13 (21%) | $45,930 ($22,446 - $69,414) | |
| 2 | 15 (24%) | $74,478 ($29,959 - $118,998) | |
| 3 | 12 (20%) | $54,486 ($20,628 -$88,343) | |
| 4 | 12 (20%) | $66,506 ($21,575 - $111,438) | |
| 5 (most advantaged) | 9 (15%) | $122,022 ($23,534 - $267,579) | |
| **LOS** | | | |
| Less than 30 days | 49 (80%) | $32,668 ($24,264 - $41,071) | |
| 30 days or more | 12 (20%) | $221,972 ($124,483 - $319,461) | |
| **Comorbidities (n)** | | | |
| 0 | 27 (44%) | $35,809 ($18,770 - $52,849) | |
| 1 | 25 (41%) | $54,708 ($32,605 - $76,812) | |
| 2 | 7 (12%) | $166,557 ($103,019 - $230,095) | |
| >3 | 2 (3%) | $381,959 ($105,075 - $868,994) | |
| **Multifocal disease (>1 site involved)** | | | |
| No | 48 (79%) | $56,998 ($25,873 - $88,123) | |
| Yes | 13 (21%) | $117,576 ($65,657- $169,494) | |
| **ID consult** | | | |
| No | 18 (30%) | $61,113 ($14,036 - $136,261) | |
| Yes | 43 (70%) | $73,590 ($51,386 - $95,794) | |

Abbreviations: Perth Children’s Hospital, PCH; *Staphylococcus aureus* bacteraemia, SAB; Accessibility/Remoteness Index of Australia, ARIA; Socio-Economic Indexes for Areas, SEIFA; length of stay, LOS; Infectious Diseases, ID

Figure 1. The distribution of community-onset SAB hospitalisation costs across the PCH Cohort

Abbreviations: Perth Children’s Hospital, PCH; Staphylococcus aureus bacteraemia, SAB; A$ Australian dollar
